# Persistent inconsistencies in patient cost variability within the French DRG classification system over the 2012–2019 period

**DOI:** 10.1186/s13561-025-00663-2

**Published:** 2025-10-30

**Authors:** Carine Milcent

**Affiliations:** 1 Paris School of Economics (PSE), PSE-ENS, 48 Boulevard Jourdan, Paris, 75012 France; 2 Centre National de la Recherche Scientifique (CNRS), PSE-ENS, 48 Boulevard Jourdan, Paris, 75012 France

**Keywords:** Cost variability drivers, Cost variation, Hospital resource costs, Cost analysis, Hospital costs, Healthcare payment, Diagnosis related groups (DRG), Bundled payment

## Abstract

This paper evaluates the effectiveness of the 2009 French Diagnosis-Related Group (DRG) classification reform, which introduced four severity levels within each DRG, ranging from low to very high, with corresponding increases in fixed-price reimbursements. Notably, the reform incorporates the Medicare Severity Diagnosis-Related Group (MS-DRG) system, first implemented in the United States in 2007, giving the French system international relevance. The French Public Health Insurance system (NHI) reimburses both public and private healthcare establishments through a DRG-based payment system. This study focuses on variations in hospital resource costs for four different health conditions. The paper begins by discussing the theoretical challenges of constructing DRG categories, particularly the trade-off between greater clinical detail (granularity) and the risk of distorting incentives for hospital efficiency. It then presents an empirical analysis of hospital resource cost variations both within and between DRGs for the same pathology or clinically meaningful group (DRG-root), using data from 2012 to 2019. Our findings suggest that a one-size-fits-all approach to severity classification is inadequate. In some cases, broader categories improve statistical validity, while in others, more granular distinctions are necessary. We conclude that a tailored, case-by-case approach is the most effective solution. Specifically, the analysis reveals significant overlap in confidence intervals for hospital resource costs across DRG severity levels, suggesting that the current classification system fails to effectively capture cost differences related to severity. Additionally, a large portion of cost variation within DRGs is driven by factors unrelated to severity, such as hospital-specific characteristics. Overall, the results underscore the need to revise the current DRG system in France in order to reduce financial discrepancies and to prevent incentives for patient selection, especially before implementing bundled payment models that include both inpatient and outpatient care.

**Supplementary Information:**

The online version contains supplementary material available at 10.1186/s13561-025-00663-2.

## Highlights

• The 2009 French DRG reform adopted a four-level severity system with corresponding increases in fixed-price ranges, based on the 2007 U.S. Medicare Severity Diagnosis-Related Group (MS-DRG) model, giving the results international relevance;
• Within a DRG, the confidence intervals for average hospital resource costs tend to overlap or merge, and the magnitude of these intervals varies randomly;
• Based on patient severity level, this revised, refined French-DRG classification does not prevent patient selection or skimming; 
• Variability in hospital costs within DRG depends on the hospital's ownership and the type of care (medical or surgical);
• This DRG cost variability study provides crucial insights to provide guidance in the design or redesign of reimbursement schemes by policymakers.

**Supplementary Information:**

The online version contains supplementary material available at 10.1186/s13561-025-00663-2.

## Introduction

In today’s healthcare landscape, bundled payments have emerged as a promising reimbursement model, attracting considerable interest from researchers [2, 3, 5, 9, 40, 42]. This approach consolidates inpatient and outpatient payments into a single, comprehensive structure tailored to patient care pathways.

In many OECD countries, inpatient care is financed through a system based on Diagnosis-Related Groups (DRGs). – a clinically structured classification framework that groups patients with similar health conditions and resource requirements [4, 7]. This system links a hospital’s case mix—the diversity and complexity of patients it treats—to the associated resource demands and costs. DRG assignment is based on factors such as diagnoses, procedures, age, sex, length of stay, and discharge status.

Research has been developed on the methods used to determine prices for inpatient care within DRGs [29, 39]. DRGs enable hospitals to gain valuable insights into their patient populations, related costs, and expected service needs, which aids in more effective resource allocation, financial planning, and care management. DRGs help ensure that hospitals can effectively balance costs and service delivery while addressing the needs of their patients. Under a regulated fixed DRG-fee system, competition shifts towards the quality of care provided rather than volume [2, 14, 20]. The DRG classification system plays a vital role in promoting equitable compensation practices among hospitals [11, 21, 26, 31, 33, 43].

Patient classification within DRGs is a dynamic process that evolves in response to changes in coding schemes, data collection methods, and medical advancements. In France, a significant reclassification of DRGs occurred in 2009 [30], aiming to improve the categorization of patients based on the severity of their condition, thus better aligning the necessary level of care [15]. As emphasized in the literature, DRG groups must be constructed to ensure both clinical and resource-based homogeneity [7]. This means that the care patients receive should be consistent, irrespective of their individual characteristics or the hospital where they are treated. As a result, hospitals are incentivized to provide the most efficient care while maintaining optimal quality within a standardized framework of practices [2, 16].^1^However, if DRG construction does not align with these goals, it may result in distorted incentives, compromising both quality of care and efficiency. Despite the significant implications of the 2009 reclassification, it remains relatively underexplored in academic research. A deeper examination of the dynamic interplay between hospital incentives and financing mechanisms offers valuable insights for healthcare regulators worldwide [22, 37].

The purpose of this paper is to assess how hospital resource costs are distributed across DRG severity levels in France. In the French healthcare system, these payments are centrally determined and do not vary at the hospital or patient level. Tariffs, which refer to the fixed payment amounts associated with each DRG, differ only by sector (public vs. private). Additionally, in three specific regions—the Paris area and two overseas territories—a uniform coefficient is applied across all DRGs and hospitals (For details, [35]). Since higher severity levels are associated with higher fixed reimbursement rates, we would expect average costs to rise accordingly. If, however, the distribution of costs overlaps across severity levels, some patients may be classified into DRGs with differing payment amounts—raising concerns about potentially arbitrary DRG assignment and misaligned provider incentives.

By analyzing the incentives shaped by the 2009 reclassification, this study reveals both intended and unintended consequences for hospital financing between 2012 and 2019. These findings contribute to a better understanding of the key considerations policymakers must consider when refining DRG classifications, to ensure that fixed-price regulations align with broader policy objectives.

Our analysis includes both a cross-sectional view for the year 2019 and a longitudinal view for the period 2012 to 2019. The hospital resource costs database includes the Electronic Health Record (EHR) data from French hospitals, along with detailed in-patient stay costs categorized by medical expenses such as staffing, logistics, medical devices, catering, and accommodation. From this data, we study four distinct types of treatment in public and private hospitals: acute myocardial infarction (AMI), stroke, cataract, and total hip replacement (THR). The PMSI (Programme de Médicalisation des Systèmes d'Information^2^) data provide access to a wealth of information. The observations are made at the level of each stay and are highly detailed regarding diagnoses, treatments, and associated costs.

In the following sections, we outline the key features of the French DRG-based payment system and the severity diagnosis-related group system. Section 3 presents our methodology while Sect. 4 discusses the databases used and presents the statistical results. In Sect. 5, we examine the results for acute myocardial infarction (AMI), allowing for comparisons with previous studies. In analyzing stroke cases, we highlight the critical role of length of stay (LOS) in determining empirical costs. Cataract surgery serves as an example of, how DRG-based payments are converted into cost-based payments. The rationale behind the severity levels within the French DRG system is further examined through an analysis of total hip Replacement (THR) cases, revealing some significant shortcomings in the system. Finally, Sect. 6 presents insights gained from a 20-year analysis of the French DRG-based prospective payment system, and Sect. 7 concludes with our key findings.

## The DRG-based payment system and the severity diagnosis-related group system

### DRG group classification in france and abroad

The number of Diagnosis-Related Groups (DRGs) varies depending on the countries where they are implemented, ranging from 400 to over 3,000. For instance, in Great Britain, Healthcare Resource Groups (HRGs)—the equivalent of DRGs—comprise approximately 2,900 groups.^3^ Similarly, Germany’s DRG system (aG-DRGs), which is based on the same principles as the French-DRG model, have around 1,300 groups.^4^ In France, the number of DRGs has grown steadily, from 2288 in 2009 to 2579 in 2013 and 2679 in 2023 (Figure B1, Appendix B, Supplementary Material).

Unlike in the U.S. system, where DRG payments are adjusted based on hospital and regional characteristics, the French system uses centrally regulated fixed tariffs. These vary by sector (public vs. private) and, in three specific regions, a uniform regional coefficient is applied to all DRGs and hospitals. No further adjustments are made (see [35] for details). 

### The logic behind the construction of DRG group homogeneity by severity level

For the DRG system to be effective, two key requirements for homogeneity must be met.

- Clinical Consistency: Patients grouped within a DRG should share similar, clinically meaningful characteristics.
- Resource Uniformity: The resources allocated to treat patients within a group should be relatively consistent, with minimal variance in hospital resource costs^5^ and utilization.

When these two conditions are met, DRGs can be considered reliable proxies for “care packages”, expected to yield optimal quality outcomes. Ideally, the DRG classification system produces clinically “meaningful” categories that align treatment needs with resource use.

If the resource allocation conditions are met, the between-DRG variance will be nearly identical to the total variance, and the within-DRG variance will be minimal. This can be validated using an indicator that measures the proportion of total cost variance attributable to within-DRG differences. A value close to zero indicates strong homogeneity within a DRG.

Conversely, a higher indicator would suggest an increase in the heterogeneity of costs for the same DRG, potentially leading to patient selection. However, if the within-DRG variance is excessively low, it could transform DRG-based payments into a procedure-fixed payment, limiting possible innovations that could increase costs. Therefore, maintaining a balance between low and zero within-DRG variances is crucial.

A large body of literature explores the advantages and drawbacks of DRG classification refinement [1, 21]. In summary, the DRG classification must find a delicate balance between ensuring an adequate number of hospital stays per group to accurately represent activity costs, while avoiding a reimbursement package that favors specific procedures. Moreover, the DRG groups must be sufficiently detailed to ensure cost homogeneity, thereby discouraging the selection of patients or procedures to the detriment of others.

Grouping patients by severity level helps to ensure a fairer measure of healthcare costs. The goal is to group patients with similar medical conditions, who also have similar resource needs. This is important because the cost of treating patients can vary greatly, and if we group patients solely based on their medical conditions, we risk selecting only those who are less expensive to treat.

This issue, known as patient selection, or skimming, has been extensively discussed in prior research [6, 27]. In their study, Horn et al. [23] assess the ability of the Severity of Illness Index to explain the variability of resource use within each DRG. Smits et al. [38] also studied the variation in resource use within diagnosis-related groups, focusing on the severity of the issue.

### The objective behind the French DRG reform

In 2009, French policymakers decided to reform the DRG classification system by introducing four severity levels within each'clinically meaningful'category, known as the DRG-root. (In this context,'clinically meaningful'refers to a subpathology that groups similar conditions for more precise classification). A French-DRG is a 6-character code, with the first five characters, referred to as the'DRG root', represents the pathology group, while the sixth digit denotes the severity level, ranging from low (1) to very high (4). As a result, for the same'clinically meaningful'category, the cost intervals were broken down in a way that aligns with the level of expenditure required to treat the patient.

Severity level is determined by an algorithm that uses information on inpatient comorbidities, complications, demographic variables, and duration of stay. The developers of the French system assume that the stochastic nature of treatment costs is accounted for within the severity levels, based on complications observed, coded, and used in the algorithm to determine the severity level. As envisioned by the French DRG system designers, the fungibility of these severity level categories is incorporated into the algorithm. For instance, a longer length of stay (LOS) alone does not result in a higher severity level.

The reform also adapted DRG classifications to accommodate low-severity patients, incorporating various modes of managed care, including day surgery and short-term medical care stays.

**Fig. 1:**
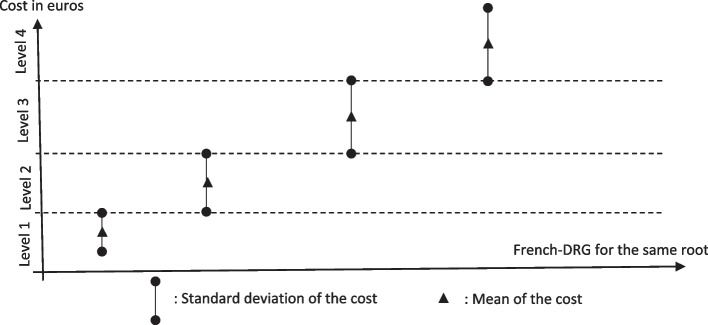
Different levels of severity for the same DRG root

The intent when designing the system was to ensure that each French DRG group corresponds to a distinct cost range that increases as it progresses in level of severity. In other words, a stay classified as low severity (level 1) should cost less than one classified under level 2, and so on, up to level 4. These cost intervals are intended to be ordered progressively according to severity level, regardless of the standard deviation's magnitude (Fig. 1).

### Theoretical considerations on variance magnitude

The cost intervals for each severity level are positioned in an ascending order on the cost scale. Taking a closer look at Fig. 1, the magnitude of the interval defined by the standard deviation can be constant or can vary, regardless of the level of severity. However, the magnitude of variance within the interval carries significant theoretical implications:

• Higher Magnitude: A greater magnitude indicates a higher variance in cost, which means a wider range of expenditure required to treat the patient. This can help anticipate patient selection and skimming behaviors.
• Smaller Magnitude: A smaller range suggests more standardized care, meaning that establishments incur the same expenses regardless of the patient. This implies that care is independent of patient characteristics, and specific costs relating to patient characteristics do not appear in the management.

As a result, an optimally defined DRG group should be neither too broad (risking cost heterogeneity and selection behavior) nor too narrow (risking inflexibility and procedural bias). The challenge lies in striking a balance that ensures both equity and efficiency.

## Methodology

### The mean cost per DRG

Simply put, as the severity levels increase, so do the costs. The severity levels in the DRG system are designed to reflect the cost of hospital stays, with higher levels requiring more financial resources. This hypothesis can be tested (Test 1) by comparing the average cost at each severity level. By doing so, we can determine whether higher severity levels correspond to greater resource utilization in patient care, on average. Assuming that the subpathology (the DRG root) is well-defined, we perform Test 1 at a refined level, the DRG-root. This test does not require cost ranges between severity levels to be entirely distinct. Instead, it tests whether, on average, higher severity levels are associated with higher resource utilization.

### Within-DRG cost variance and econometric cost models

For any given year, **hospital status** and pathology, we can compute the average cost per DRG across all hospitals. This cost is denoted as *Cg* with *g* being a DRG for the same pathology or, when refined, a DRG-root (a “clinically meaningful” group).

Variance provides a comprehensive measure of dispersion, but its sensitivity to extreme values can be a drawback. For this reason, the interquartile range is sometimes preferred. However, relying on the interquartile range would have prevented us from calculating the within-variance portion of the total variance. This portion refers to the dispersion due to cost differences within the same DRG, as opposed to variance resulting from cost differences linked to varying severity levels. To address this, we ran a sensitivity test, by removing the first and last quartiles. The results remained unchanged. In addition, a box-and-whisker plot for each DRG-root is included in the supplementary materials to visually represent cost dispersion. The variance in the cost of stays can be analyzed as follows:

$$V\left(Cg,i\right)=V\left(Cg.\right)+V(Cg,i-Cg.)$$

Index *i* indicates the stay. The total cost variance, *V(Cg,i)* equals the sum of.

• Between-DRG variance *V(Cg.)*: due to differences in average costs between DRGs.
• Within-DRG variance, *V(Cg,i—Cg.)*: due to cost differences between stays within the same DRG.

Between 2012 and 2019, hospital pricing was based on a fixed price per DRG. This means that hospitals with the same distribution of stays/DRGs would have the same operating budget for their activities. DRGs are assumed to meet two conditions: first, patients are assumed to be perfectly homogeneous, and second, hospitals are assumed to treat patients identically. This hypothesis can be tested by calculating the indicator that measures the proportion of cost variance due to differences in average costs within DRGs, Test 2.

$$(V(Cg,i-Cg.)/V(Cg,i))$$

• An indicator close to 0 means that if all patients of a certain DRG were identical and hospitals treated them in the same way, the total variance and between-DRG variance would be equal, while the within-DRG variance would be zero *(V(Cg,i—Cg.)* = *0)*. This indicates that the hypothesis is verified;
• If the indicator is close to 1, it suggests that the hypothesis is not supported, indicating a high degree of cost heterogeneity within the same DRG.

### Econometric models

For the cost of the stay, the skewness and kurtosis tests indicate a non-normal distribution for each of the four conditions, with *p* < 0.0001 for each test. We then consider a logarithm of the total cost model.

*C*_*i,g,h,t*_ the logarithm of the cost of stay *i* for *DRG g* admitted to hospital *h*, year *t.*

*• LOS*_*i,g,h,t*_ the log of the length of stay^6^*i* for *DRG g* admitted to hospital *h*, year *t*
*• X*_*i,g,h,t*_ patient’s characteristics for *DRG g* admitted to hospital *h*, year *t*
• Age, sex
• Diagnoses
• Comorbidities
• Complications
• α_*g*_ DRG dummies
*• Y*_*t*_ year dummies
• β_*h*_ hospital dummies
*• u*^*m*^_*i,g,h,t*_ error terms, with *m* superscript for model with *m* = *1, …, 4*.

The estimated models are of the form,

1. *C*_*i,g,h,t*_ = α_*g*_ + *Y*_*t*_ + *u*^*1*^_*i,g,h,t*_
2. *C*_*i,g,h,t*_ = α_*g*_ + *Y*_*t*_ + β_*h*_ + *u*^*2*^_*i,g,h,t*_
3. *C*_*i,g,h,t*_ = α_*g*_ + *Y*_*t*_ + β_*h*_ + γ*X *_*i,g,t*_ + *u*^*3*^_*i,g,h,t*_
4. *C*_*i,g,h,t*_ = α_*g*_ + *Y*_*t*_ + β_*h*_ + γ*X *_*i,g,t*_ + θ*LOS*_*i,g,h,t*_ + *u*^*4*^_*i,g,h,t*_

The explanatory variables for total cost of stay are not independent of each other. Thus, the additional variance explained by the addition of a new explanatory variable depends on the composition of variables already present in the model. The greater the value (in %) of the R^2^, the more explanatory the model, i.e. the set of explanatory variables predicts the total cost of the stay for the treatment in question.

The dataset consists of panel data. For each model, the Hausman test yields a highly significant result (*p* < 0.001), indicating that a fixed effects model is preferred over a random effects specification. Accordingly, we estimate an OLS regression with hospital-fixed effects to control for time-invariant unobserved heterogeneity across hospitals.

## Database

### The cost database

The ENCC (Échelle Nationale des Coûts à Méthodologie Commune) is a medico-economic database that collects Electronic Health Records (EHR) as well as the actual costs of hospital stays broken down by medical expenditure categories, such as staff, logistics, medical devices, catering, and accommodation. This ENCC database is compiled annually by ATIH (Agence Technique de l’Information sur l’Hospitalisation), a public agency of the French Health Ministry.

Participation in the ENCC is voluntary, with hospitals opting to participate in order to better understand the cost structure of their medical activities. Both public and private institutions can take part, provided their cost accounting systems have been certified as reliable by ATIH.

The ENCC employs comprehensive cost accounting methods that include all types of expenses, including medical device expenditures paid outside the DRG fee, as well as remuneration for doctors not covered by DRG payments in the private sector. This inclusive approach ensures that all costs are accounted for, providing a clear and accurate representation of the actual hospital expenditures. By incorporating these costs into the accounting system, the ENCC is able to provide a more complete financial picture, which is essential for informed decision-making and effective resource allocation.

A detailed breakdown of costs per DRG along with their respective share of total costs, is available on the ATIH website: https://www.scansante.fr/applications/enc-mco.

The medico-economic data used in this study are costs per French DRG and serve as the basis for the ENCC database. Stays for four distinct health conditions were selected using the DRG root from 2012 to 2019.

• Acute myocardial infarction (AMI): 05M04, 05M16, 05K05, 05K06, 05K21
• Stroke: 01M30 and 01M31
• Cataract: 02C05 and 02C12
• Total hip replacement (THR): emergency THR 08C47 and planned THR 08C48

### Cost database and French context

In France, hospital staff management differs significantly between public and private hospitals. In public hospitals, most staff members are civil servants, which grants them job security, but their salaries are regulated by the central government and determined according to a national grading system.

In contrast, private hospitals operate under a different framework: hospital staff are salaried employees, while physicians are typically either self-employed or salaried.

Although staffing costs are often higher in urban areas compared to rural ones, these differences are not directly accounted for by policymakers when setting the fixed tariffs for hospital-administered care. To account for regional disparities in local price levels, a regional geographic coefficient is applied, based on the regional consumer price index.

ATIH processes cost data separately for the public and private sectors to produce a preliminary tariff and develops two distinct cost scales—one for the public sector and one for the private sector—based on the reported cost information.

## Results

### Preliminary statistical results

Table 1 presents descriptive statistics for the four health conditions considered. Notably, the data show that patients receiving care in 2019 were, on average, older than those treated in 2012. Additionally, there has been a significant decrease in the length of hospital stays over time. Furthermore, the proportion of patients discharged home following treatment increased markedly in 2019 compared to 2012.

**Table 1:** Descriptive statistics per disorder (THP, Cataract, AMI, Stroke)

		Total Hip Replacement (THR)				Cataract					
Years		2019		2012		2019			2012		
Observations		17′062		16′808		70′571			71′244		
		Mean	Standard error	Mean	Standard error	Mean	Standard error		Mean	Standard error	
Sex (%)		59,00	-	60,23	-	57,41	-		58,61	0,493	
Age (years)		72,59	12,62	71,95	13,37	73,35	9,4		73,25	11,42	
Length of stay (days)		5,29	5,65	9,28	5.,6	0,04	0,31		0,20	0,74	
Root 1	Cost, public sector (€)	9 209,01	5369,39	8954,09	5316,63	1758,06	544		1625,10	562,41	
	Cost, private sector (€)	6847,88	4439,59	7283,96	2436,31	1065,18	134,59		1230,95	188,61	
Root 2	Cost, public sector (€)	6934,72	2692,61	7716,63	2960,43	2823,10	1032,49		2709,60	1618,46	
	Cost, private sector (€)	5129,94	1110,22	6119,43	1027,56	2032,95	1477,73		1583,60	244,01	
**Hospital admission**											
	From another hospital	1,93	-	1,85	-	0,05	-		0,15	-	
	From home	97,96	-	97,80	-	99,89	-		99,78	-	
**Hospital discharge**											
	To another unit	3,34	-	6,79	-	0,02	-		0,03	-	
	To another hospital	27,70	-	37,45	-	0,09	-		0,04	-	
	To home	68,09	-	54,71	-	99,86	-		99,91	-	
		Acute Myocardial Infarction (AMI)				Stroke					
Years		2019		2012		2019			2012		
Observations		38′881		41′746		15′627			19′485		
		Mean	Standard error	Mean	Standard error	Mean		Standard error	Mean		Standard error
Sex(%)		29,60	-	27,12	-	46,64		-	48,56		-
Age (years)		69,55	12,9	67,91	13,62	72,09		16,02	70.80		17,3
Length of stay (days)		3,68	4,71	4,59	5,72	9,63		11,78	10,56		12,35
Root 1	Cost, public sector (€)	7003,35	5177,80	7095,57	5998,97	6534,27		7496,64	7406,39		8532,04
	Cost, private sector (€)	6079,25	2566,65	6227,24	2523,34	4983,64		6234,42	6167,58		7544,8
Root 2	Cost, public sector (€)	5230,75	4916,47	5362,50	4524,77	4869,26		9037,33	5020,71		8697,15
	Cost, private sector (€)	4586,77	2496,24	4678,14	2238,80	4759,11		13,467,72	5091,42		11,466,07
Root 3	Cost, public sector (€)	24,492,52	7476,67	32,792,75	12,042,31	-		-	-		-
	Cost, private sector (€)	23,320,49	5123,92	29,116,62	10,878,05	-		-	-		-
Root 4	Cost, public sector (€)	4512,17	5431,45	5268.47	6617.29	-		-	-		-
	Cost, private sector (€)	3688,04	2108,50	4204,64	2828,54	-		-	-		-
Root 5	Cost, public sector (€)	2772,61	3391,32	2607,88	3688,10	-		-	-		-
	Cost, private sector (€)	1763,77	1556,03	1867,67	1828,36	-		-	-		-
**Hospital admission (%)**											
	From another hospital	9,47	-	9,05	-	13,11		-	7,95		-
	From home	89,07	-	89,43	-	86,38		-	91,75		-
**Hospital discharge (%)**											
	To another unit	0,32	-	0,61	-	5,77		-	8,07		-
	To another hospital	13,21	-	13,11	-	35,48		-	28,36		-
	To home	83,51	-	82,47	-	50,11		-	53,78		-
	In-patient death	1,57	-	2,38	-	8,27		-	9,69		-

*Source*: Database ENC 2012–2019 per health condition

Root 1 and Root 2: with THR, 08C48 and 08C47; with Cataract, 02C05 and 02C12; with stroke, 01M30 and 01M31; Root 1 to 5: with AMI, 05K05, 05K06, 05K21, 05M04, and 05M16

The mean cost is calculated while controlling for inflation and changes in time

The number and composition of hospitals reporting cost data to the ENCC vary slightly from year to year. These data serve as the official basis for DRG tariff setting by the regulator, and the resulting tariffs are applied uniformly across all hospitals within each sector. Results are reported separately for the years 2012 and 2019

Appendix A in the supplementary material contains Tables A1 to A8, which expand the observation count by detailing observations by severity level within roots

After adjusting for inflation and changes in databases, the cost per DRG root was higher for the public sector than the private one. Moreover, we do not observe a systematic increase in costs over time – for instance, stroke-related costs show a decrease. It is important to note that these costs exclude certain medical devices, a limitation that applies to both public and private sectors.^7^ These exclusions should be considered when analyzing hospital resource costs in order to obtain a more comprehensive understanding of the expenses involved. For instance, in the public sector, the cost for 01M30 DRG-root (stroke) was approximately 7,400€ in 2012 but 6,500€ in 2019; in the private sector, the same DRG-root cost 6.200€ in 2012 and 5,000€ in 2019.

A statistical analysis of patient discharge outcomes indicates that the majority, approximately 90%, returned home after receiving medical treatment. However, this rate varied significantly depending on the nature of the ailment. For instance, patients who underwent cataract surgery were much more likely to return home compared to those who suffered from a stroke, where only 50% were able to do so.

The analysis further revealed a positive trend in the number of THP (total hip replacement) patients discharged home after undergoing surgery [34].

### From theory to empirics: construction of DRG groups (Test 1)

**Fig. 2:**
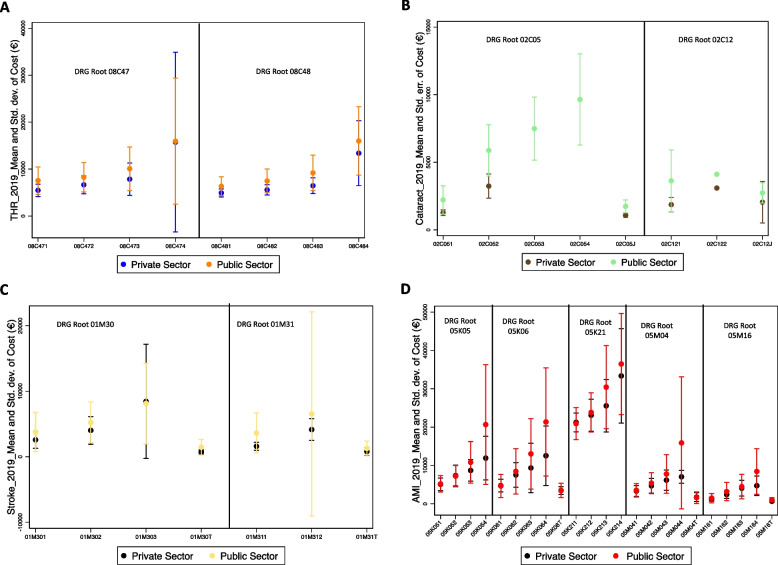
Stay’s cost per DRG and sector (2019) _ Mean and Standard deviation. Source: Base ENC, 2012–2019. Note: Each code represents a DRG, which stands for the root associated with a specific severity level. The mean is represented by a dot, and the standard deviation is shown by a vertical segment that defines the interval. Besides, a one-way ANOVA was conducted to examine whether there are significant differences in the means of the DRG groups based on their DRG-root classification. For THR, two DRG roots, 08C47 and 08C48, each has four severity levels. For cataract, two DRG roots, 02C05 and 02C12, and each root has four levels of severity. The last digit with"J"in the French DRG represents a low-severity patient whose care is managed through day surgery. Under DRG-root 02C05, no statistically significant differences were found between the means of the respective DRGs. For AMI, DRG roots are 05K05, 05K06, 05K21, 05M04 and 05M16, and each root has four levels of severity. The last digit with the letter"T"in the French DRG indicates a low-severity patient who receives medical care for a short stay of two days or less. Within the private sector, no statistically significant differences were observed between the means of the following DRGs under DRG-root 01M05: 01M054 and 01M055; and under DRG-root 01M30: 01M301 and 01M30T. For stroke, two DRG roots are 01M30 and 01M31. The last digit with the letter"T"in the French DRG indicates a low-severity patient who receives medical care for a short stay of two days or less. Within the private sector, no statistically significant differences were observed between the means of the following DRGs under DRG-root 01M30: 01M301 and 01M302; and under DRG-root 05M16: 05M16T and 05M161, 05M163 and 05M164. Similarly, within the public sector, no statistically significant differences were observed between the means of the following DRGs under DRG-root 05M16: 05M16T and 05M161

Figure 2 shows the mean and standard deviation of stay costs per severity level for a given DRG root in both the public and private sectors.

Revisiting the comparison of the theoretical and empirical frameworks, we focus on surgical procedures, namely THR and cataract surgery.

For THR, we analyzed the light-orange dots and segments representing public sector costs. Our analysis found that the cost range for low severity cases (level 1), as defined by the standard deviation, overlaps with the cost range for moderate severity cases (level 2). This overlap pattern continues across all subsequent severity levels, up to and including the highest level (level 4). A similar outcome was observed for the private sector.

A notable outlier is the standard deviation for French-DRG 08C474, corresponding to emergency THR associated with the highest severity level, which is particularly high.

For cataract procedures, most activities are concentrated at the lower severity levels, typically managed through overnight stays or day surgery. This suggests the possible need to refine the DRG classification for this procedure, assuming the ENCC database accurately reflects overall hospital activity. An ANOVA test comparing means was conducted for DRG-root 02C12 (lens operations with trabeculectomy), where the equality of means test (*Test 1*) indicated that hospital resource costs are comparable across the different DRGs. However, the severity level here could not be considered due to the lack of observations (see Supplementary Materials, Appendix A, Tables A3 and A4).

Focusing exclusively on the mean cost of DRG-root 02C05 (lens operations with or without vitrectomy) provides empirical evidence that aligns with the theoretical model (Fig. 2B). However, the very limited number of observations for the moderate severity level undermines the robustness of this result.

When treating stroke, medical care differs significantly from procedures such as cataract surgery or THR. Stroke cases are classified into two French-DRG roots, namely 01M30 and 01M31. Figure 2C presents the results for both private (black) and public (yellow) sectors. Stroke severity is divided into four levels, each corresponding to a DRG group for very short stays. However, the practical outcomes are at odds with the theoretical framework, raising questions about the accuracy of creating French-DRG clusters based on roots. In addition, for the private sector, an ANOVA test for stroke revealed that the mean hospital resource costs between low (Level 1) and moderate (Level 2) severity levels are not significantly different at the 1% level.

For acute myocardial infarction (AMI), another ANOVA test (Test 1), showed no significant difference in mean hospital resource costs between the high and very high severity levels of DRG-root 05M16 in the private sector. However, the very small number of observations weakens the reliability of this result. Overall, the findings indicate that the confidence intervals for average costs tend to overlap or converge across severity levels within each DRG-root, regardless of the sector (Fig. 2D). This pattern undermines the assumption that severity level is a consistent predictor of resource utilization within DRG roots.

Upon examining the points and segments, it becomes clear that when categorizing conditions with French-DRGs according to the level of severity, the practical implementation of cost interval construction deviates from theoretical reasoning. This inconsistency appears across all four health conditions, regardless of the French-DRG root being considered. This raises doubts regarding the multiplication of the DRG root into four severity levels and indicates the need to reevaluate the DRG classification. A more streamlined classification system, with fewer subdivisions, may be more appropriate.

Lastly, Appendix B in the supplementary materials includes a detailed presentation of variation in length of stay by DRG (Figure B2).

### Within-DRG variance in stays (Test 2)

Table 2 presents the results of the variance decomposition analysis, distinguishing between within-DRG and between-DRG variance. For total hip replacement (THR), stroke, and cataract, within-DRG variance accounts for 80% of the entire variance (Test 2).

**Table 2:** Within-DRG cost variance, 2012–2020

Years	2012	2013	2014	2015	2016	2017	2018	2019
	THR							
	Public sector							
No. of stays	11,312	11,592	11,124	11,016	10,642	12,132	11,653	9297
(%) Within-DRG variance	85,77%	89,27%	83,09%	84,90%	77,04%	74,14%	81,60%	78,15%
	Private Sector							
No. of stays	5496	6381	7067	6573	6092	7509	8769	7765
(%) Within-DRG variance	79,48%	83,53%	80,47%	84,80%	81,77%	72,83%	76,68%	74,79%
	Stroke							
	Public sector							
No. of stays	19,044	19,426	17,927	19,119	16,703	18,289	15,852	15,292
(%) Within-DRG variance	81,18%	79,57%	74,19%	74,65%	57,94%	79,90%	77,79%	75,31%
	Private Sector							
No. of stays	441	408	446	239	267	276	343	335
(%) Within-DRG variance	Not enough observations available							
	Cataract							
	Public sector							
No. of stays	37,969	37,832	33,183	40,668	31,212	40,059	37,969	44,153
(%) Within-DRG variance	78,76%	66,09%	78,44%	77,87%	84,09%	77,31%	64,46%	83,37%
	Private Sector							
No. of stays	33,275	35,750	40,177	41,908	36,310	47,494	56,032	26,418
(%) Within-DRG variance	88,83%	86,13%	94,27%	84,32%	82,19%	93,19%	85,88%	77,96%
	AMI							
	Public sector							
No. of stays	33,460	34,383	31,511	33,560	33,010	35,464	34,432	29,973
(%) Within-DRG variance	41,20%	39,53%	30,89%	39,47%	29,00%	37,90%	35,71%	37,71%
	Private Sector							
No. of stays	8286	8399	7691	7398	9349	9730	10,474	8908
(%) Within-DRG variance	30,03%	23,10%	22,49%	13,47%	20,15%	17,48%	13,56%	14,64%

*Source*: Database ENC 2012–2019 per health condition

Root 1 and Root 2: with THR, 08C48 and 08C47; with Cataract, 02C05 and 02C12; with stroke, 01M30 and 01M31; Root 1 to 5: with AMI, 05K05, 05K06, 05K21, 05M04, and 05M16

The number and composition of hospitals reporting cost data to the ENCC vary slightly from year to year. These data serve as the official basis for DRG tariff setting by the regulator, and the resulting tariffs are applied uniformly across all hospitals within each sector. Results are reported separately for each year

When analyzing acute myocardial infarction (AMI), the cost range for high-severity DRGs includes the average costs associated with very short-stays, as well as those of low and moderate severity levels, in both public and private sectors.

Previous studies by Dormont and Milcent [11], and Milcent [31] provide an evolutionary view of within-DRG variance in France. Dormont and Milcent's paper presented the situation before the introduction of DRG-based payment system,^8^ from 1994 to 1995, while Milcent [31] presented the first French DRG-based payment system assessments for the period 2006–2011. Here, we focus on within-DRG variance over the period 2012 to 2019.

For reasons of comparability, we have opted for an analysis based on comparable French-DRGs over time.

The results show that within-DRG variance in 2012 is comparable to 2011 estimates from Milcent’ [31], which was around 40% for the public sector and 30% for the private sector. For a longitudinal view from 2012 to 2019, the within-DRG variance decreases for both sectors. It suggests a decline in within-DRG heterogeneity.

### Explained variance of total costs

We then conducted a residual analysis to understand how much of the variation in hospital resource costs can be explained by different factors.

Jacobs et al. [24] reviewed the determinants that account for variability in hospital costs. Continuing in this line, we developed a set of models to analyze the costs associated with each health condition (Table 3). For each one, we began with Model 1, which considers only French-DRG indicators to explain the total cost. This model provides a basis for comparison with more complex models incorporating additional variables.

**Table 3:** Logarithm of the hospital resource total costs estimation model, 2012–2019

Total Hip Replacement—THR									
	Public sector					Private sector			
Model	−1	−2	−3	−4	Model	−1	−2	−3	−4
Observations	88,768	88,768	88,768	88,768	Observations	55,652	55,652	55,652	55,652
Adjusted R^2^	0.2537	0.3341	0.3639	0.7713	Adjusted R^2^	0.2519	0.4043	0.4351	0.7229
**Stroke**									
	Public sector					Private sector			
Model	−1	−2	−3	−4	Model	−1	−2	−3	−4
Observations	141,652	141,652	141,652	141,652	Observations	2,755	2,755	2,755	2,755
Adjusted R^2^	0.5223	0.5588	0.5840	0.8499	Adjusted R^2^	0.5998	0.7410	0.7550	0.9189
**Cataract**									
	Public sector					Private sector			
Model	−1	−2	−3	−4	Model	−1	−2	−3	−4
Obs	285,310	285,310	285,310	285,310	Obs	335,099	335,099	335,099	335,099
Adjusted R^2^	0.1474	0.5112	0.5148	0.5560	Adjusted R^2^	0.1977	0.5083	0.5117	0.5260
**Acute Myocardial Infarction – AMI**									
	Public sector					Private sector			
Model	−1	−2	−3	−4	Model	−1	−2	−3	−4
Observations	265,793	265,793	265,793	265,793	Observations	70,235	70,235	70,235	70,235
Adjusted R^2^	0.6901	0.7121	0.7153	0.8090	Adjusted R^2^	0.7239	0.7329	0.7344	0.7959

(1) (%) R2 of the model explained by DRG indicators and time dummies

(2) (%) R2 of the model explained by DRG indicators, times dummies, and hospital fixed effects

(3) (%) R2 of the model explained by DRG indicators, times dummies, hospital fixed effects, and patient casemix excluding length of stay

(4) (%) R2 of the model explained by DRG indicators, times dummies, hospital fixed effects, and patient casemix including length of stay

The variance explained by each model is not simply additive – each one accounts for the remaining variance from the previous models. Therefore, the variance explained by each model may overlap, and the total variance explained by all models cannot be directly summed. When presenting results as incremental, it's important to keep in mind that this can lead to overinterpretation of the results [12, 13, 31].

After analyzing the results of Model 1, we move on to more comprehensive models that take into account multiple factors that affect the cost of treatment. Model 4, presented in Eq. (4), is the complete model. It considers a range of variables beyond French-DRG indicators, including patient demographics, diagnosis, comorbidities, and complications. By presenting multiple models for each health condition, we aim to provide a more nuanced understanding of the factors that impact treatment costs. Comparing the results of different models helps us to identify the most significant variables in order to make more informed decisions about healthcare policy and resource allocation.

Our complete model, Model 4, explains cost variability: between 50% (for cataract) and 90% (for stroke).

As shown in Table 3, length of stay (LOS) was found to be a significant factor in explaining cost variability for stroke patients, as well as THR in-patients, between 2012 and 2019.

#### By health condition

The cost of total hip replacement (THR) can vary considerably, with the primary factor being the length of stay (LOS). When including LOS in Model 3, which already accounts for patient case-mix, hospital characteristics, and French-DRG, LOS leads to a 30 to 40% increase in total cost variability, depending on the sector. Therefore, Model 4 explains 72 to 77% of the total cost variance. The French-DRG system similarly explains the cost variability in the private sector (25.2%) compared to the public sector (25.4%).

Cataract surgery is primarily provided by the private sector in France. The French-DRG alone explains one-fifth of the cost variability (Model 1) in the private sector, compared to 14.7% in the public sector. In Model 3, the results between sectors are comparable, and when controlling for LOS, the results remain unchanged. This is because of the generally short LOS for patients across all severity levels, except for DRG-root 02C05 in the public sector, where more complex medical conditions or vulnerabilities are typically admitted to public healthcare facilities. In Model 4, we show that LOS does not explain additional cost differences between healthcare facilities.

Our analysis of cost variations related to AMI (acute myocardial infarction) found that the French-DRG system is effective for this pathology, accounting for 69% to 72% of the cost variability. Length of stay is also a contributing factor, though not a major one. It explains an additional 5.8% of the variance in the private sector and 8.8% in the public sector. These results support findings in the literature [11, 31] though with a lower level of explanatory power.

## Discussion

Since the introduction of DRGs, a substantial body of literature has emerged to investigate the drivers of cost variability in hospital care, as noted by Pirson et al. [36]. The 2009 DRG classification revision introduced severity-based subcategories, dividing each DRG root into four levels that correspond to lump-sum payments. As illustrated in Fig. 1, hospital resource costs are expected to increase with severity level, as the DRG classification system is designed to align higher severity levels with greater associated costs. Building on prior studies (Horn et al., [23, 38]) by evaluating severity indices and resource use within DRGs, this paper examines cost variability.

We have analyzed four different health conditions over the period 2012–2019: total hip replacement (THR), cataract surgery, stroke, and acute myocardial infarction (AMI), with each of these allowing us to investigate the variability of costs within the French Diagnosis Related Group (DRG) framework from different perspectives. These conditions span a spectrum of care types: surgical (THR and cataract), medical (stroke), and cardiac pathology (AMI). They differ in complexity, sector distribution and resource use. Cataract operations, for example, which are primarily performed in the private sector, do not require overnight stays, unlike THR operations, which typically require hospitalization.^9^ Stroke care is mainly managed by the public sector, while AMI is a pathology that has been historically studied for DRG cost variability [10, 11, 31].

The results reveal a clear misalignment between the empirical observations and the theoretical framework in three out of four health conditions, with the only exception being DRG-root 02C05 for stroke, which displays a distinct pattern (Fig. 2B). The analysis of within-DRG variances by health condition and year (Table 2) reveals a consistent pattern of approximately 80% variance throughout the observed period, except for AMI.

Such findings raise concerns regarding the classification system’s approach of subdividing the root into four levels of severity. This means that the risk is concentrated within the DRG group, rather than between DRGs of the same pathology or the same clinically meaningful group (e.g., DRG-root). The risk is not shared properly between the payer and the provider. Additionally, we found that the cost range for a certain level of severity intersects with the cost range for the subsequent level of severity. After identifying sources of cost variability, healthcare providers can redesign the existing care pathways of a subgroup of patients to reduce costs, as has been underlined by Keel [25]. Additionally, providers can avoid expensive combinations of treatments for individual patients and instead turn to lower-cost alternatives. To address this issue, a re-evaluation of the DRG classification is recommended, potentially streamlining it to a smaller number of severity levels proportional to the root categories.

In the case of surgical procedures (such as THR procedures and, in the private sector, cataract procedures) we observe cases where the total cost variance is almost negligible, regardless of the severity level, except at very high severity levels. This finding aligns with Meng et al.’s [28] study, which analyzed the effects of DRG-based payment systems compared to cost-based payment on inpatient healthcare utilization. The aim of refining the classification is to homogenize costs for the same DRG [21]. However, the more refined the classification, the closer this pricing method comes to fixed-cost pricing rather than to fixed-tariff pricing based on average costs.

As a result, the regulatory authorities become responsible for decisions on patient care methods. They no longer pay for the outcomes of care, but for the care process. What matters to the patient is not the means used to provide care, but quite simply the outcome, by whatever the means. In this sense, payment systems should prioritize outcomes and allow providers flexibility in determining how care is delivered, as long as quality standards are met.

The ownership structure of healthcare facilities also plays a crucial role in shaping patient care management, as emphasized by Feess et al. [17]) and further demonstrated in the French context by Milcent [32]. This dynamic is particularly evident in our analysis of total hip replacement, where incorporating the patient’s case mix into the model—already explained by DRG classifications (comparing Model 1 to Model 2)—accounts for 15% of cost variability in the private sector, compared to just 8% in the public sector. These findings suggest that DRG classifications, when combined with patient case mix, reveal varying levels of cost variance depending on the ownership model. This applies to all the health conditions studied, except for cataract, and indicates sector-specific dynamics.

The significant impact of length of stay (LOS) on health outcomes, particularly for stroke is a key factor in explaining cost variability across both public and private healthcare sectors, as highlighted by Feess et al. [17]. When the original Medicare Diagnosis-Related Group (DRG) system was introduced for this public program—and subsequently adopted by private payers—one notable result was a dramatic reduction in LOS. This suggests that the prior cost-based reimbursement system had incentivized unnecessarily prolonged hospital stays. However, Struijs [41], notes that LOS, along with other indicators of resource utilization, is influenced not only by patient and treatment characteristics, but also by the quality of care provided, including any shortcomings in that care. To improve fairness and efficiency, reimbursement schemes should adjust for patient and treatment characteristics, rather than relying solely on LOS as the primary determinant.

Some examples, such as cataracts with DRG-root 02C05, illustrate how the multiplication of DRG codes can lead to greater homogeneity within these groups. These findings suggest that subgroups within a DRG-root can be validated, enhancing specificity and accuracy in patient classification. However, these findings cannot be generalized, as a limited number of observations for higher severity levels jeopardizes the results.

These four pathologies represent only a fraction of what is encompassed by the French DRG classification, thus it is reasonable to consider that for other pathologies, the results might differ. Our choice of these particular pathologies was guided by their frequent examination in scientific literature. Therefore, while there is no reason to believe our findings would deviate significantly from global trends, the possibility cannot be entirely ruled out.

A limitation of our study is that the PMSI dataset records hospitalizations rather than individual patients. Consequently, we could not assess other metrics that might indicate disease severity, such as readmission rates or post-discharge outcomes. While this may influence the case-mix analyzed in this study, it does not impact the severity levels as defined by the DRG classification, since the codes used in the algorithm are standardized by the regulatory authority.

The data source is based on a sample of voluntary hospitals, representing about 10% of all hospitals in France—both public and private. Participation in the ENCC (National Cost Study) varies year to year, making it a constant challenge for ATIH to recruit and retain hospitals in the study. While there may be incentives to over-report costs, hospitals cannot expect a direct benefit from doing so, as the reported costs are averaged across all institutions within a given sector. For any impact to occur, it would require a collective decision from all participating hospitals. Moreover, the tariffs derived from these cost data are used to regulate care across the entire hospital system. Therefore, such a coordinated manipulation across facilities is an unlikely scenario.

The representativeness of these data—particularly in reflecting actual hospital costs nationwide—remains a concern raised by French auditing and oversight agencies. Nevertheless, these cost data serve as the foundation for calculating hospital care tariffs across the entire healthcare system, both public and private. Thus, although the representativeness of the data is debated outside the circle of system developers advising Health Ministry policymakers, the ENCC remains the official national standard for hospital cost estimation and pricing used in the annual tariff-setting process.

## Conclusion

Using data from 2012 to 2019, this study evaluates the effectiveness and originality of the 2009 French DRG classification reform, which was designed to finance both public and private healthcare establishments. The reform introduced a sub-classification within each DRG root group, adding four levels of severity, ranging from low to very high, with corresponding increases in fixed-price payments. Notably, the reform incorporated the Medicare Severity Diagnosis-Related Group (MS-DRG) system, first implemented in the United States in 2007, giving the results international relevance.

Our study examines the relationship between DRG changes, case mix variations, and hospital reimbursement. We have determined that a one-size-fits-all approach to severity classification may be counterproductive. For some clinically meaningful groups, a coarser classification could provide better statistical validity, whereas for others, a more detailed approach may be appropriate. Based on our findings, we conclude that a case-by-case approach is the most suitable overall.

A functional reimbursement model should be adjusted based on case-mix to discourage cherry-picking and to encourage efficiency improvements, such as avoiding unnecessary spending. Importantly, our results reveal that the confidence intervals for average hospital resource costs per level of severity within the same clinically meaningful group—DRG root—often overlap or merge. Moreover, the magnitude of these intervals varies randomly, without any clear pattern related to severity levels. As a result, constructing refined French-DRG groups in this way does not prevent patient selection or skimming.

The study of the relationships between DRGs, cost, and quality in the U.S. dates back over three decades [8]. According to Gluckman et al. [19], in a U.S. hospitalization cohort study, DRG shifts were associated with at least $1.2 billion in increased payments. The overlap in costs between different levels of severity creates an incentive to shift cases to higher severity levels. This underscores the need to appropriately refine the Diagnosis-Related Group (DRG) classification by severity level.

DRG classifications vary significantly from one country to another, both in their design and in their ability to explain hospital costs, as notably highlighted by the work of Geissler et al. [18]. While several studies have focused on DRG classification systems, to our knowledge none has specifically analyzed the contributions of the MS-DRG model, introduced in the United States in 2007. Our study is, therefore, one of the first to examine the distribution of hospital costs within MS-DRG categories, showing in particular that, although the average cost increases with severity, the standard deviations overlap considerably, revealing persistent cost heterogeneity.

In summary, our findings collectively highlight the urgent need to improve the French DRG severity model to address substantial discrepancies in preventive measures, particularly concerning patient selection or skimming practices. This refinement is crucial before transitioning to a bundled payment system that integrates both in-patient and out-patient services. Ultimately, the necessity of refining these mechanisms is key to fostering a more equitable, efficient, and outcome-oriented healthcare system.

## Supplementary Information

Supplementary Material 1.

## Data Availability

Data Availability Statement The data supporting this study's funding are available from the French Agency of Hospital Information (ATIH) Digital. Restrictions apply to the availability of these data used under the license for this study. Data Protection Authority Data used for this study are reported to the National Data Protection Authority (CNIL number 2019-100001-166-140).

## Footnotes

1 This theoretical model assumes two points: firstly, that competition is expressed, i.e., that the number of establishments allows this competition. Secondly, patients have information – in the sense of having access to the medical information on the theoretical frame of reference for appropriate care and the empirical practices of the hospital they wish to visit – as well as the knowledge needed to understand the medical information provided.

2 PMSI which translates to “Medicalization Program for Information Systems” standardizes hospital data for diagnoses and treatments. Integrated into EMRs (Electronic Medical Records), it improves resource management, billing, and patient care efficiency.

3 https://mtrconsult.com/news/202223-hrg4-local-payment-grouper-came-force-april-1-2022-england, consulted 04/05/2024.

4 https://reimbursement.institute/glossar/ag-drg-system/, consulted 04/05/2024.

5 In the remainder of this paper, the term"cost"will refer specifically to the hospital resource costs.

6 Since the model explains the log of cost, it is more appropriate to use the log of duration as an explanatory variable to maintain a linear relationship and allow for an elasticity interpretation. To test linearity, we regressed the log of cost on duration and duration squared. The squared term was not significant. We also considered the R^2^ and the residual plots. This analysis was conducted for all 4 health conditions.

7 Certain medical devices are reimbursed in addition to DRG tariffs. The Health Ministry establishes and publishes an annual list of these devices. This regulation applies to both the public and private sectors.

8 In France, the DRG-based payment system is called T2A – Tarification à l'Activité.

9 Since 2013, there has been a significant increase in day surgery admissions, although the period observed only goes up to 2019.</
