# Supplementary material for: Persistent inconsistencies in patient cost variability within the French DRG classification system over the 2012–2019 period
Source: Health Econ Rev. 2025 Oct 30;15:91. doi: 10.1186/s13561-025-00663-2 (PMC12574288; doi:10.1186/s13561-025-00663-2)
Supplement: Supplementary file 1 — Supplementary Material 1. [file 13561_2025_663_MOESM1_ESM.docx]

# APPENDIX A

## Total Hip Replacement _ THR

Tableau A1 _ THR, Year 2019, **Public Sector**: Detailed information on Cost and LOS per French-DRG

| DRG-Root | Variable | N | Mean | Std. Dev. | Q25 | Q50 | Q75 | D90 |
| --- | --- | --- | --- | --- | --- | --- | --- | --- |
|  |  |  |  |  |  |  |  |  |
| 08C47 | Total cost | 3625 | 9209,01 | 5369,39 | 6513,70 | 8030,29 | 10287,95 | 13752,61 |
|  | LOS | 3625 | 9,40 | 6,82 | 6,00 | 8,00 | 11,00 | 16,00 |
|  |  |  |  |  |  |  |  |  |
| 08C48 | Total cost | 5672 | 6934,72 | 2692,61 | 5513,04 | 6324,05 | 7629,03 | 9570,62 |
|  | LOS | 5672 | 4,25 | 3,16 | 2,00 | 4,00 | 5,00 | 7,00 |
|  |  |  |  |  |  |  |  |  |
| DRG-Root | Variable | N | Mean | Std. Dev. | Q25 | Q50 | Q75 | D90 |
|  |  |  |  |  |  |  |  |  |
| 08C471 | Total cost | 780 | 7552,45 | 2922,62 | 5841,94 | 7003,62 | 8657,20 | 10483,28 |
|  | LOS | 780 | 6,24 | 3,15 | 4,00 | 6,00 | 7,50 | 10,00 |
|  |  |  |  |  |  |  |  |  |
| 08C472 | Total cost | 1414 | 8254,41 | 3137,01 | 6267,06 | 7561,06 | 9383,75 | 11792,44 |
|  | LOS | 1414 | 8,19 | 4,37 | 5,00 | 7,00 | 10,00 | 13,00 |
|  |  |  |  |  |  |  |  |  |
| 08C473 | Total cost | 1192 | 10074,86 | 4653,04 | 7291,85 | 8984,06 | 11516,56 | 15002,73 |
|  | LOS | 1192 | 11,07 | 6,95 | 7,00 | 9,00 | 13,00 | 19,00 |
|  |  |  |  |  |  |  |  |  |
| 08C474 | Total cost | 239 | 15944,68 | 13436,76 | 9098,84 | 12894,34 | 18678,56 | 26162,96 |
|  | LOS | 239 | 18,50 | 13,49 | 10,00 | 15,00 | 24,00 | 34,00 |
|  |  |  |  |  |  |  |  |  |
| 08C481 | Total cost | 3745 | 6331,45 | 2011,11 | 5160,14 | 5913,63 | 6967,00 | 8408,63 |
|  | LOS | 3745 | 3,26 | 1,97 | 2,00 | 3,00 | 4,00 | 6,00 |
|  |  |  |  |  |  |  |  |  |
| 08C482 | Total cost | 1381 | 7476,84 | 2541,15 | 6028,50 | 6913,72 | 8274,47 | 9940,12 |
|  | LOS | 1381 | 5,28 | 2,48 | 3,00 | 5,00 | 7,00 | 8,00 |
|  |  |  |  |  |  |  |  |  |
| 08C483 | Total cost | 505 | 9190,23 | 3788,01 | 6979,01 | 8287,80 | 9913,87 | 12873,64 |
|  | LOS | 505 | 7,82 | 5,15 | 5,00 | 7,00 | 9,00 | 14,00 |
|  |  |  |  |  |  |  |  |  |
| 08C484 | Total cost | 41 | 15996,12 | 7289,06 | 10479,33 | 14311,18 | 20656,06 | 24633,88 |
|  | LOS | 41 | 15,88 | 8,99 | 10,00 | 13,00 | 19,00 | 26,00 |

Tableau A2 _ THR, Year 2019, **Private Sector**: Detailed information on Cost and LOS per French-DRG

| DRG-Root | Variable | N | Mean | Std. Dev. | Q25 | Q50 | Q75 | D90 |
| --- | --- | --- | --- | --- | --- | --- | --- | --- |
|  |  |  |  |  |  |  |  |  |
| 08C47 | Total cost | 877 | 6847,88 | 4439,60 | 5162,90 | 6015,69 | 7372,96 | 9441,15 |
|  | LOS | 877 | 8,95 | 14,28 | 5,00 | 7,00 | 10,00 | 14,00 |
|  |  |  |  |  |  |  |  |  |
| 08C48 | Total cost | 6888 | 5129,94 | 1110,22 | 4395,66 | 4936,42 | 5601,44 | 6347,22 |
|  | LOS | 6888 | 3,53 | 2,43 | 2,00 | 3,00 | 4,00 | 6,00 |
|  |  |  |  |  |  |  |  |  |
| DRG-Root | Variable | N | Mean | Std. Dev. | Q25 | Q50 | Q75 | D90 |
|  |  |  |  |  |  |  |  |  |
| 08C471 | Total cost | 297 | 5476,51 | 1318,24 | 4636,37 | 5289,76 | 6006,31 | 6925,95 |
|  | LOS | 297 | 5,48 | 2,79 | 4,00 | 5,00 | 7,00 | 9,00 |
|  |  |  |  |  |  |  |  |  |
| 08C472 | Total cost | 328 | 6647,43 | 1914,01 | 5380,35 | 6207,90 | 7405,08 | 9246,78 |
|  | LOS | 328 | 8,21 | 4,55 | 5,00 | 7,00 | 10,00 | 13,00 |
|  |  |  |  |  |  |  |  |  |
| 08C473 | Total cost | 224 | 7846,69 | 3448,54 | 5812,92 | 6846,14 | 8740,01 | 11426,19 |
|  | LOS | 224 | 11,43 | 8,77 | 6,00 | 9,00 | 13,00 | 21,00 |
|  |  |  |  |  |  |  |  |  |
| 08C474 | Total cost | 28 | 15751,71 | 19135,61 | 7001,79 | 8704,93 | 17914,52 | 31857,31 |
|  | LOS | 28 | 34,68 | 69,03 | 9,00 | 14,50 | 32,00 | 65,00 |
|  |  |  |  |  |  |  |  |  |
| 08C481 | Total cost | 5277 | 4926,30 | 853,22 | 4287,59 | 4776,63 | 5389,47 | 6044,79 |
|  | LOS | 5277 | 2,96 | 1,81 | 2,00 | 3,00 | 4,00 | 5,00 |
|  |  |  |  |  |  |  |  |  |
| 08C482 | Total cost | 1297 | 5578,87 | 1098,15 | 4870,71 | 5397,33 | 6054,35 | 6801,48 |
|  | LOS | 1297 | 4,90 | 2,11 | 3,00 | 4,00 | 6,00 | 7,00 |
|  |  |  |  |  |  |  |  |  |
| 08C483 | Total cost | 303 | 6455,15 | 1674,26 | 5325,99 | 6068,40 | 7044,95 | 8326,98 |
|  | LOS | 303 | 6,99 | 3,67 | 5,00 | 6,00 | 8,00 | 12,00 |
|  |  |  |  |  |  |  |  |  |
| 08C484 | Total cost | 11 | 13382,26 | 6907,46 | 6474,25 | 12373,97 | 21570,53 | 23242,70 |
|  | LOS | 11 | 20,18 | 16,25 | 6,00 | 17,00 | 28,00 | 38,00 |

## Cataract

Tableau A3 _ Cataract, Year 2019, **Public Sector**: Detailed information on Cost and LOS per French-DRG

| DRG-Root | Variable | N | Mean | Std. Dev. | Q25 | Q50 | Q75 | D90 |
| --- | --- | --- | --- | --- | --- | --- | --- | --- |
|  |  |  |  |  |  |  |  |  |
| 02C05 | Total cost | 26060 | 1758,07 | 544,71 | 1503,95 | 1695,39 | 1928,64 | 2220,17 |
|  | LOS | 26060 | 0,05 | 0,44 | 0,00 | 0,00 | 0,00 | 0,00 |
|  |  |  |  |  |  |  |  |  |
| 02C12 | Total cost | 358 | 2823,10 | 1032,50 | 2283,58 | 2751,38 | 3136,79 | 3815,71 |
|  | LOS | 358 | 0,21 | 0,95 | 0,00 | 0,00 | 0,00 | 1,00 |
|  |  |  |  |  |  |  |  |  |
| DRG | Variable | N | Mean | Std. Dev. | Q25 | Q50 | Q75 | D90 |
|  |  |  |  |  |  |  |  |  |
| 02C051 | Total cost | 829 | 2211,66 | 1048,88 | 1698,40 | 2001,15 | 2346,00 | 3225,43 |
|  | LOS | 829 | 1,24 | 0,96 | 1,00 | 1,00 | 1,00 | 2,00 |
|  |  |  |  |  |  |  |  |  |
| 02C052 | Total cost | 21 | 5879,46 | 1903,38 | 4675,96 | 5613,91 | 6549,50 | 7747,90 |
|  | LOS | 21 | 6,05 | 2,42 | 4,00 | 5,00 | 9,00 | 9,00 |
|  |  |  |  |  |  |  |  |  |
| 02C053 | Total cost | 8 | 7490,73 | 2331,80 | 5773,42 | 7773,75 | 8893,12 | 11187,15 |
|  | LOS | 8 | 12,25 | 4,80 | 9,00 | 13,00 | 16,00 | 18,00 |
|  |  |  |  |  |  |  |  |  |
| 02C054 | Total cost | 3 | 9645,81 | 3373,57 | 5981,51 | 10333,12 | 12622,79 | 12622,79 |
|  | LOS | 3 | 14,33 | 11,85 | 7,00 | 8,00 | 28,00 | 28,00 |
|  |  |  |  |  |  |  |  |  |
| 02C05J | Total cost | 25199 | 1736,95 | 475,59 | 1503,57 | 1680,12 | 1902,92 | 2179,23 |
|  | LOS | 25199 | 0,00 | 0,00 | 0,00 | 0,00 | 0,00 | 0,00 |
|  |  |  |  |  |  |  |  |  |
| 02C121 | Total cost | 35 | 3626,94 | 2280,25 | 2245,89 | 3124,57 | 3801,47 | 6237,77 |
|  | LOS | 35 | 2,03 | 2,33 | 1,00 | 1,00 | 2,00 | 5,00 |
|  |  |  |  |  |  |  |  |  |
| 02C122 | Total cost | 1,00 | 4108,42 | , | 4108,42 | 4108,42 | 4108,42 | 4108,42 |
|  | LOS | 1,00 | 3,00 | , | 3,00 | 3,00 | 3,00 | 3,00 |
|  |  |  |  |  |  |  |  |  |
| 02C12J | Total cost | 322,00 | 2731,74 | 742,23 | 2283,58 | 2700,44 | 3064,79 | 3728,53 |
|  | LOS | 322,00 | 0,00 | 0,00 | 0,00 | 0,00 | 0,00 | 0,00 |

Tableau A4 _ Cataract, Year 2019, **Private Sector**: Detailed information on Cost and LOS per French-DRG

| DRG-Root | Variable | N | Mean | Std. Dev. | Q25 | Q50 | Q75 | D90 |
| --- | --- | --- | --- | --- | --- | --- | --- | --- |
|  |  |  |  |  |  |  |  |  |
| 02C05 | Total cost | 43713 | 1065,19 | 134,59 | 988,31 | 1050,65 | 1094,92 | 1209,72 |
|  | LOS | 43713 | 0,02 | 0,17 | 0,00 | 0,00 | 0,00 | 0,00 |
|  |  |  |  |  |  |  |  |  |
| 02C12 | Total cost | 440 | 2032,95 | 1477,74 | 1611,52 | 2118,16 | 2249,85 | 2388,24 |
|  | LOS | 440 | 0,09 | 0,33 | 0,00 | 0,00 | 0,00 | 0,00 |
|  |  |  |  |  |  |  |  |  |
| DRG-Root | Variable | N | Mean | Std. Dev. | Q25 | Q50 | Q75 | D90 |
|  |  |  |  |  |  |  |  |  |
| 02C051 | Total cost | 1022 | 1265,71 | 215,48 | 1148,97 | 1218,36 | 1337,02 | 1527,43 |
|  | LOS | 1022 | 1,02 | 0,16 | 1,00 | 1,00 | 1,00 | 1,00 |
|  |  |  |  |  |  |  |  |  |
| 02C052 | Total cost | 5 | 3235,09 | 893,78 | 2627,88 | 3173,15 | 3605,64 | 4533,51 |
|  | LOS | 5 | 5,80 | 2,59 | 5,00 | 5,00 | 6,00 | 10,00 |
|  |  |  |  |  |  |  |  |  |
| 02C05J | Total cost | 42686 | 1060,13 | 125,80 | 988,31 | 1048,41 | 1088,06 | 1185,44 |
|  | LOS | 42686 | 0,00 | 0,00 | 0,00 | 0,00 | 0,00 | 0,00 |
|  |  |  |  |  |  |  |  |  |
| 02C121 | Total cost | 33 | 1867,43 | 528,60 | 1420,88 | 1579,35 | 2422,57 | 2493,79 |
|  | LOS | 33 | 1,06 | 0,35 | 1,00 | 1,00 | 1,00 | 1,00 |
|  |  |  |  |  |  |  |  |  |
| 02C122 | Total cost | 1 | 3092,40 | . | 3092,40 | 3092,40 | 3092,40 | 3092,40 |
|  | LOS | 1 | 3,00 | . | 3,00 | 3,00 | 3,00 | 3,00 |
|  |  |  |  |  |  |  |  |  |
| 02C12J | Total cost | 406 | 2043,79 | 1529,65 | 1618,49 | 2118,16 | 2247,89 | 2281,69 |
|  | LOS | 406 | 0,00 | 0,00 | 0,00 | 0,00 | 0,00 | 0,00 |

## Acute Myocardial Infarction _ AMI

Tableau A5 _ AMI, Year 2019, **Public Sector**: Detailed information on Cost and LOS per French-DRG

| DRG-Root | Variable | N | Mean | | Std. Dev. | | Q25 | | Q50 | | Q75 | | D90 | | |
| --- | --- | --- | --- | --- | --- | --- | --- | --- | --- | --- | --- | --- | --- | --- | --- |
|  |  |  | |  | |  | |  | |  | |  | |  | |
| 05K05 | Total cost | 8174 | | 7003,35 | | 5177,80 | | 4380,36 | | 5764,51 | | 8008,82 | | 11130,43 | |
|  | LOS | 8174 | | 5,19 | | 5,24 | | 2,00 | | 4,00 | | 6,00 | | 9,00 | |
|  |  |  | |  | |  | |  | |  | |  | |  | |
| 05K06 | Total cost | 14723 | | 5230,75 | | 4916,47 | | 2936,47 | | 3886,85 | | 5583,80 | | 8958,67 | |
|  | LOS | 14723 | | 2,70 | | 4,10 | | 1,00 | | 2,00 | | 2,00 | | 5,00 | |
|  |  |  | |  | |  | |  | |  | |  | |  | |
| 05K21 | Total cost | 1662 | | 24492,52 | | 7476,67 | | 20587,66 | | 22878,47 | | 26547,72 | | 31974,83 | |
|  | LOS | 1662 | | 6,82 | | 5,59 | | 4,00 | | 5,00 | | 8,00 | | 13,00 | |
|  |  |  | |  | |  | |  | |  | |  | |  | |
| 05M04 | Total cost | 4291 | | 4512,17 | | 5431,45 | | 1931,46 | | 3375,17 | | 5487,65 | | 8371,27 | |
|  | LOS | 4291 | | 4,65 | | 6,12 | | 1,00 | | 3,00 | | 6,00 | | 11,00 | |
|  |  |  | |  | |  | |  | |  | |  | |  | |
| 05M16 | Total cost | 1123 | | 2772,61 | | 3391,32 | | 929,42 | | 1639,70 | | 3146,57 | | 6223,76 | |
|  | LOS | 1123 | | 4,15 | | 5,80 | | 1,00 | | 2,00 | | 5,00 | | 10,00 | |
|  |  |  | |  | |  | |  | |  | |  | |  | |
| DRG-Root | Variable | N | Mean | | Std. Dev. | | Q25 | | Q50 | | Q75 | | D90 | |  |
|  |  |  | |  | |  | |  | |  | |  | |  | |
| 05K051 | Total cost | 4202 | | 5205,46 | | 2163,07 | | 3801,63 | | 4732,19 | | 6111,11 | | 7895,70 | |
|  | LOS | 4202 | | 2,97 | | 1,77 | | 2,00 | | 2,00 | | 4,00 | | 5,00 | |
|  |  |  | |  | |  | |  | |  | |  | |  | |
| 05K052 | Total cost | 2941 | | 7218,44 | | 2811,33 | | 5254,46 | | 6599,42 | | 8522,69 | | 10920,01 | |
|  | LOS | 2941 | | 5,83 | | 2,99 | | 4,00 | | 5,00 | | 7,00 | | 9,00 | |
|  |  |  | |  | |  | |  | |  | |  | |  | |
| 05K053 | Total cost | 727 | | 10817,71 | | 5421,92 | | 7185,52 | | 9474,17 | | 12960,53 | | 17215,50 | |
|  | LOS | 727 | | 10,10 | | 6,45 | | 6,00 | | 8,00 | | 13,00 | | 18,00 | |
|  |  |  | |  | |  | |  | |  | |  | |  | |
| 05K054 | Total cost | 304 | | 20651,67 | | 15623,07 | | 10643,89 | | 15116,51 | | 24817,75 | | 35408,13 | |
|  | LOS | 304 | | 18,02 | | 14,71 | | 9,00 | | 14,00 | | 22,00 | | 36,00 | |
|  |  |  | |  | |  | |  | |  | |  | |  | |
| 05K061 | Total cost | 7081 | | 4653,33 | | 2995,51 | | 3063,21 | | 3945,90 | | 5195,94 | | 7092,72 | |
|  | LOS | 7081 | | 2,30 | | 0,96 | | 2,00 | | 2,00 | | 2,00 | | 3,00 | |
|  |  |  | |  | |  | |  | |  | |  | |  | |
| 05K062 | Total cost | 1463 | | 8460,47 | | 5938,04 | | 5134,56 | | 6778,04 | | 9693,79 | | 13689,26 | |
|  | LOS | 1463 | | 5,68 | | 3,57 | | 3,00 | | 4,00 | | 7,00 | | 10,00 | |
|  |  |  | |  | |  | |  | |  | |  | |  | |
| 05K063 | Total cost | 690 | | 13029,26 | | 9170,22 | | 7273,55 | | 10112,54 | | 15301,22 | | 24279,21 | |
|  | LOS | 690 | | 11,27 | | 8,35 | | 6,00 | | 8,00 | | 14,00 | | 21,00 | |
|  |  |  | |  | |  | |  | |  | |  | |  | |
| 05K064 | Total cost | 195 | | 21342,19 | | 14098,18 | | 11371,86 | | 17885,20 | | 26497,63 | | 42122,79 | |
|  | LOS | 195 | | 19,73 | | 12,49 | | 10,00 | | 16,00 | | 26,00 | | 37,00 | |
|  |  |  | |  | |  | |  | |  | |  | |  | |
| 05K06T | Total cost | 5294 | | 3500,67 | | 1855,73 | | 2481,42 | | 3138,51 | | 4025,84 | | 5301,08 | |
|  | LOS | 5294 | | 0,67 | | 0,47 | | 0,00 | | 1,00 | | 1,00 | | 1,00 | |
|  |  |  | |  | |  | |  | |  | |  | |  | |
| 05K211 | Total cost | 409 | | 20906,74 | | 4176,74 | | 19058,50 | | 20839,24 | | 22472,21 | | 24544,19 | |
|  | LOS | 409 | | 3,56 | | 1,97 | | 2,00 | | 3,00 | | 4,00 | | 6,00 | |
|  |  |  | |  | |  | |  | |  | |  | |  | |
| 05K212 | Total cost | 966 | | 23818,88 | | 5119,20 | | 20910,16 | | 23023,65 | | 26039,96 | | 29238,78 | |
|  | LOS | 966 | | 6,23 | | 3,23 | | 4,00 | | 5,00 | | 7,00 | | 10,00 | |
|  |  |  | |  | |  | |  | |  | |  | |  | |
| 05K213 | Total cost | 218 | | 30416,92 | | 10814,20 | | 23096,27 | | 28327,66 | | 35007,86 | | 42143,11 | |
|  | LOS | 218 | | 12,31 | | 8,21 | | 7,00 | | 9,00 | | 16,00 | | 22,00 | |
|  |  |  | |  | |  | |  | |  | |  | |  | |
| 05K214 | Total cost | 69 | | 36460,59 | | 13192,61 | | 26881,07 | | 32917,99 | | 42534,89 | | 58415,30 | |
|  | LOS | 69 | | 17,09 | | 10,17 | | 9,00 | | 15,00 | | 22,00 | | 32,00 | |
|  |  |  | |  | |  | |  | |  | |  | |  | |
| 05M041 | Total cost | 1292 | | 3530,58 | | 1742,13 | | 2360,80 | | 3142,17 | | 4259,63 | | 5765,53 | |
|  | LOS | 1292 | | 3,11 | | 1,70 | | 2,00 | | 2,00 | | 4,00 | | 5,00 | |
|  |  |  | |  | |  | |  | |  | |  | |  | |
| 05M042 | Total cost | 1136 | | 5382,28 | | 2744,28 | | 3540,74 | | 4798,65 | | 6585,49 | | 8720,69 | |
|  | LOS | 1136 | | 6,17 | | 3,44 | | 4,00 | | 5,00 | | 8,00 | | 11,00 | |
|  |  |  | |  | |  | |  | |  | |  | |  | |
| 05M043 | Total cost | 477 | | 7777,07 | | 5037,73 | | 4509,85 | | 6327,32 | | 9241,17 | | 13909,63 | |
|  | LOS | 477 | | 10,60 | | 7,20 | | 6,00 | | 9,00 | | 13,00 | | 19,00 | |
|  |  |  | |  | |  | |  | |  | |  | |  | |
| 05M044 | Total cost | 193 | | 15881,59 | | 17229,53 | | 6647,98 | | 10289,51 | | 17390,88 | | 32201,30 | |
|  | LOS | 193 | | 17,16 | | 15,34 | | 8,00 | | 13,00 | | 20,00 | | 30,00 | |
|  |  |  | |  | |  | |  | |  | |  | |  | |
| 05M04T | Total cost | 1193 | | 1601,97 | | 1525,50 | | 751,76 | | 1132,44 | | 1933,97 | | 3127,24 | |
|  | LOS | 1193 | | 0,48 | | 0,50 | | 0,00 | | 0,00 | | 1,00 | | 1,00 | |
|  |  |  | |  | |  | |  | |  | |  | |  | |
| 05M161 | Total cost | 523 | | 1476,72 | | 1222,68 | | 801,89 | | 1145,50 | | 1695,16 | | 2685,71 | |
|  | LOS | 523 | | 1,98 | | 1,39 | | 1,00 | | 2,00 | | 2,00 | | 4,00 | |
|  |  |  | |  | |  | |  | |  | |  | |  | |
| 05M162 | Total cost | 186 | | 3189,68 | | 2379,61 | | 1802,54 | | 2398,28 | | 3654,00 | | 5327,66 | |
|  | LOS | 186 | | 5,19 | | 3,36 | | 3,00 | | 4,00 | | 6,00 | | 8,00 | |
|  |  |  | |  | |  | |  | |  | |  | |  | |
| 05M163 | Total cost | 129 | | 4490,37 | | 3188,65 | | 2498,41 | | 3496,43 | | 5348,19 | | 8520,78 | |
|  | LOS | 129 | | 8,07 | | 6,79 | | 5,00 | | 6,00 | | 9,00 | | 14,00 | |
|  |  |  | |  | |  | |  | |  | |  | |  | |
| 05M164 | Total cost | 118 | | 8443,83 | | 5936,29 | | 4465,05 | | 6483,87 | | 10599,54 | | 16825,19 | |
|  | LOS | 118 | | 13,69 | | 9,46 | | 8,00 | | 10,00 | | 14,00 | | 28,00 | |
|  |  |  | |  | |  | |  | |  | |  | |  | |
| 05M16T | Total cost | 167 | | 1032,37 | | 598,81 | | 684,87 | | 819,92 | | 1100,85 | | 1987,65 | |
|  | LOS | 167 | | 0,00 | | 0,00 | | 0,00 | | 0,00 | | 0,00 | | 0,00 | |

Tableau A6 _ AMI, Year 2019, **Private Sector**: Detailed information on Cost and LOS per French-DRG

| DRG-Root | Variable | | N | Mean | | Std. Dev. | | Q25 | | | Q50 | | Q75 | | D90 | |
| --- | --- | --- | --- | --- | --- | --- | --- | --- | --- | --- | --- | --- | --- | --- | --- | --- |
|  |  |  | | |  | |  | |  |  | |  | |  | |  |
| 05K05 | Total cost | | 1101 | | 6079,25 | | 2566,65 | | 4334,09 | 5551,17 | | 7174,28 | | 9290,87 | |  |
|  | LOS | | 1101 | | 3,87 | | 2,56 | | 2,00 | 3,00 | | 5,00 | | 7,00 | |  |
|  |  | |  | |  | |  | |  |  | |  | |  | |  |
| 05K06 | Total cost | | 6350 | | 4586,77 | | 2496,24 | | 3165,58 | 4006,34 | | 5138,43 | | 6829,64 | |  |
|  | LOS | | 6350 | | 2,03 | | 2,36 | | 1,00 | 2,00 | | 2,00 | | 4,00 | |  |
|  |  | |  | |  | |  | |  |  | |  | |  | |  |
| 05K21 | Total cost | | 865 | | 23320,49 | | 5123,92 | | 20755,89 | 21853,46 | | 24593,01 | | 28312,51 | |  |
|  | LOS | | 865 | | 6,53 | | 3,80 | | 5,00 | 6,00 | | 7,00 | | 10,00 | |  |
|  |  | |  | |  | |  | |  |  | |  | |  | |  |
| 05M04 | Total cost | | 473 | | 3688,04 | | 2108,50 | | 2190,64 | 3438,65 | | 4778,03 | | 6526,63 | |  |
|  | LOS | | 473 | | 3,68 | | 3,35 | | 2,00 | 3,00 | | 5,00 | | 7,00 | |  |
|  |  | |  | |  | |  | |  |  | |  | |  | |  |
| 05M16 | Total cost | | 119 | | 1763,77 | | 1556,03 | | 860,07 | 1327,88 | | 2127,14 | | 3664,92 | |  |
|  | LOS | | 119 | | 2,53 | | 3,05 | | 1,00 | 2,00 | | 3,00 | | 6,00 | |  |
|  |  |  | | |  | |  | |  |  | |  | |  | |  |
| DRG-Root | Variable | | N | Mean | | Std. Dev. | | Q25 | | | Q50 | | Q75 | | D90 | |
|  |  |  | | |  | |  | |  |  | |  | |  | |  |
| 05K051 | Total cost | | 673 | | 5 071,13 | | 1 629,75 | | 3 908,54 | 4 796,35 | | 5 838,24 | | 7 207,76 | |  |
|  | LOS | | 673 | | 2,81 | | 1,56 | | 2,00 | 2,00 | | 4,00 | | 5,00 | |  |
|  |  | |  | |  | |  | |  |  | |  | |  | |  |
| 05K052 | Total cost | | 366 | | 7 383,18 | | 2 701,94 | | 5 643,62 | 6 936,76 | | 8 719,59 | | 10 592,71 | |  |
|  | LOS | | 366 | | 5,11 | | 2,46 | | 3,00 | 4,00 | | 6,00 | | 8,00 | |  |
|  |  | |  | |  | |  | |  |  | |  | |  | |  |
| 05K053 | Total cost | | 50 | | 8 703,01 | | 2 790,91 | | 6 416,20 | 8 010,24 | | 10 795,95 | | 12 217,39 | |  |
|  | LOS | | 50 | | 7,30 | | 3,38 | | 5,00 | 6,00 | | 9,00 | | 13,00 | |  |
|  |  | |  | |  | |  | |  |  | |  | |  | |  |
| 05K054 | Total cost | | 12 | | 11 916,01 | | 5 688,40 | | 7 708,93 | 10 417,60 | | 14 395,77 | | 18 965,87 | |  |
|  | LOS | | 12 | | 10,92 | | 5,43 | | 6,50 | 9,00 | | 14,50 | | 17,00 | |  |
|  |  | |  | |  | |  | |  |  | |  | |  | |  |
| 05K061 | Total cost | | 2728 | | 4 753,28 | | 1 652,23 | | 3 711,79 | 4 481,76 | | 5 302,50 | | 6 455,17 | |  |
|  | LOS | | 2728 | | 2,28 | | 0,77 | | 2,00 | 2,00 | | 2,00 | | 3,00 | |  |
|  |  |  | | |  | |  | |  |  | |  | |  | |  |
| 05K062 | Total cost | | 576 | | 7 526,67 | | 3 200,54 | | 5 386,75 | 6 728,16 | | 9 001,41 | | 11 208,09 | |  |
|  | LOS | | 576 | | 4,60 | | 2,97 | | 3,00 | 4,00 | | 5,00 | | 7,00 | |  |
|  |  | |  | |  | |  | |  |  | |  | |  | |  |
| 05K063 | Total cost | | 162 | | 9 362,45 | | 6 436,47 | | 6 086,97 | 7 793,01 | | 10 181,62 | | 14 800,55 | |  |
|  | LOS | | 162 | | 8,41 | | 5,75 | | 5,00 | 6,00 | | 9,00 | | 17,00 | |  |
|  |  | |  | |  | |  | |  |  | |  | |  | |  |
| 05K064 | Total cost | | 35 | | 12 533,20 | | 7 737,08 | | 7 140,81 | 9 730,98 | | 14 990,13 | | 19 388,66 | |  |
|  | LOS | | 35 | | 13,86 | | 9,18 | | 7,00 | 12,00 | | 17,00 | | 22,00 | |  |
|  |  | |  | |  | |  | |  |  | |  | |  | |  |
| 05K06T | Total cost | | 2849 | | 3 463,77 | | 1 021,29 | | 2 789,60 | 3 224,70 | | 3 928,06 | | 4 677,30 | |  |
|  | LOS | | 2849 | | 0,78 | | 0,42 | | 1,00 | 1,00 | | 1,00 | | 1,00 | |  |
|  |  | |  | |  | |  | |  |  | |  | |  | |  |
| 05K211 | Total cost | | 171 | | 21 240,55 | | 2 450,59 | | 19 926,56 | 20 996,04 | | 22 121,98 | | 23 582,02 | |  |
|  | LOS | | 171 | | 4,33 | | 1,74 | | 2,00 | 5,00 | | 5,00 | | 6,00 | |  |
|  |  |  | | |  | |  | |  |  | |  | |  | |  |
| 05K212 | Total cost | | 557 | | 23 101,91 | | 4 160,87 | | 20 794,37 | 21 950,76 | | 24 708,02 | | 27 887,06 | |  |
|  | LOS | | 557 | | 6,41 | | 2,63 | | 5,00 | 6,00 | | 7,00 | | 10,00 | |  |
|  |  | |  | |  | |  | |  |  | |  | |  | |  |
| 05K213 | Total cost | | 115 | | 25 552,51 | | 6 820,78 | | 21 475,67 | 23 421,49 | | 27 166,03 | | 33 574,88 | |  |
|  | LOS | | 115 | | 8,61 | | 4,57 | | 5,00 | 8,00 | | 10,00 | | 13,00 | |  |
|  |  | |  | |  | |  | |  |  | |  | |  | |  |
| 05K214 | Total cost | | 22 | | 33 354,11 | | 12 304,37 | | 24 922,62 | 29 430,51 | | 36 447,33 | | 58 225,80 | |  |
|  | LOS | | 22 | | 15,82 | | 10,95 | | 9,00 | 14,00 | | 17,00 | | 38,00 | |  |
|  |  | |  | |  | |  | |  |  | |  | |  | |  |
| 05M041 | Total cost | | 172 | | 3 325,01 | | 1 397,24 | | 2 165,34 | 3 234,06 | | 4 086,68 | | 5 261,80 | |  |
|  | LOS | | 172 | | 3,03 | | 1,72 | | 2,00 | 2,00 | | 4,00 | | 5,00 | |  |
|  |  | |  | |  | |  | |  |  | |  | |  | |  |
| 05M042 | Total cost | | 160 | | 4 697,60 | | 1 890,60 | | 3 271,67 | 4 498,01 | | 5 842,23 | | 6 996,46 | |  |
|  | LOS | | 160 | | 4,95 | | 2,50 | | 3,00 | 4,00 | | 6,00 | | 9,00 | |  |
|  |  |  | | |  | |  | |  |  | |  | |  | |  |
| 05M043 | Total cost | | 33 | | 6 177,47 | | 2 627,40 | | 4 150,99 | 6 234,25 | | 7 679,11 | | 9 554,01 | |  |
|  | LOS | | 33 | | 9,42 | | 5,78 | | 5,00 | 8,00 | | 12,00 | | 16,00 | |  |
|  |  | |  | |  | |  | |  |  | |  | |  | |  |
| 05M044 | Total cost | | 5 | | 7 044,85 | | 1 675,16 | | 6 568,78 | 6 670,84 | | 8 004,84 | | 9 218,18 | |  |
|  | LOS | | 5 | | 10,40 | | 5,46 | | 7,00 | 9,00 | | 12,00 | | 19,00 | |  |
|  |  | |  | |  | |  | |  |  | |  | |  | |  |
| 05M04T | Total cost | | 103 | | 1 765,47 | | 1 158,13 | | 677,88 | 1 592,37 | | 2 670,08 | | 3 078,69 | |  |
|  | LOS | | 103 | | 0,64 | | 0,48 | | - | 1,00 | | 1,00 | | 1,00 | |  |
|  |  | |  | |  | |  | |  |  | |  | |  | |  |
| 05M161 | Total cost | | 65 | | 1 228,23 | | 521,37 | | 812,15 | 1 229,78 | | 1 483,13 | | 1 835,46 | |  |
|  | LOS | | 65 | | 1,60 | | 0,75 | | 1,00 | 1,00 | | 2,00 | | 2,00 | |  |
|  |  | |  | |  | |  | |  |  | |  | |  | |  |
| 05M162 | Total cost | | 16 | | 2 419,33 | | 1 026,68 | | 1 640,83 | 2 190,49 | | 3 039,20 | | 3 756,88 | |  |
|  | LOS | | 16 | | 4,00 | | 1,51 | | 3,00 | 3,00 | | 4,50 | | 6,00 | |  |
|  |  |  | | |  | |  | |  |  | |  | |  | |  |
| 05M163 | Total cost | | 11 | | 4 079,57 | | 2 070,17 | | 3 017,51 | 3 590,29 | | 4 596,14 | | 4 867,72 | |  |
|  | LOS | | 11 | | 6,27 | | 2,69 | | 4,00 | 6,00 | | 7,00 | | 9,00 | |  |
|  |  | |  | |  | |  | |  |  | |  | |  | |  |
| 05M164 | Total cost | | 7 | | 4 720,42 | | 2 524,41 | | 2 939,66 | 3 934,69 | | 6 324,84 | | 9 713,76 | |  |
|  | LOS | | 7 | | 9,14 | | 6,79 | | 5,00 | 6,00 | | 10,00 | | 24,00 | |  |
|  |  | |  | |  | |  | |  |  | |  | |  | |  |
| 05M16T | Total cost | | 20 | | 671,27 | | 310,57 | | 320,97 | 791,36 | | 934,05 | | 977,63 | |  |
|  | LOS | | 20 | | - | | - | | - | - | | - | | - | |  |

## Stroke

Tableau A7 _ Stroke, Year 2019, **Public Sector**: Detailed information on Cost and LOS per French-DRG

| DRG-Root | Variable | | N | | Mean | | Std. Dev. | | Q25 | | Q50 | | Q75 | | D90 | |
| --- | --- | --- | --- | --- | --- | --- | --- | --- | --- | --- | --- | --- | --- | --- | --- | --- |
| 01M30 | Total cost | 13956 | | 6534,27 | | 7496,64 | | 2795,94 | | 4665,18 | | 7658,58 | | 12495,15 | |  |
|  | LOS | 13956 | | 9,88 | | 11,40 | | 3,00 | | 7,00 | | 12,00 | | 21,00 | |  |
|  |  |  | |  | |  | |  | |  | |  | |  | |  |
| 01M31 | Total cost | 1336 | | 4869,26 | | 9037,33 | | 1095,69 | | 2866,86 | | 6097,67 | | 10533,00 | |  |
|  | LOS | 1336 | | 6,62 | | 11,09 | | 1,00 | | 3,00 | | 9,00 | | 16,00 | |  |
|  |  |  | |  | |  | |  | |  | |  | |  | |  |
| DRG-Root | Variable | | N | | Mean | | Std. Dev. | | Q25 | | Q50 | | Q75 | | D90 | |
|  |  |  | |  | |  | |  | |  | |  | |  | |  |
| 01M301 | Total cost | 3235 | | 3770,63 | | 3004,12 | | 2144,98 | | 3134,14 | | 4619,73 | | 6596,95 | |  |
|  | LOS | 3235 | | 4,78 | | 3,41 | | 2,00 | | 4,00 | | 6,00 | | 9,00 | |  |
|  |  |  | |  | |  | |  | |  | |  | |  | |  |
| 01M302 | Total cost | 3519 | | 5241,16 | | 3112,76 | | 3126,14 | | 4533,59 | | 6356,49 | | 8972,77 | |  |
|  | LOS | 3519 | | 8,18 | | 5,56 | | 4,00 | | 7,00 | | 10,00 | | 15,00 | |  |
|  |  |  | |  | |  | |  | |  | |  | |  | |  |
| 01M303 | Total cost | 4343 | | 8134,02 | | 6157,57 | | 4488,47 | | 6518,07 | | 9747,21 | | 14548,94 | |  |
|  | LOS | 4343 | | 13,56 | | 10,37 | | 7,00 | | 11,00 | | 16,00 | | 25,00 | |  |
|  |  |  | |  | |  | |  | |  | |  | |  | |  |
| 01M304 | Total cost | 1474 | | 15749,75 | | 15453,29 | | 6657,37 | | 11106,75 | | 18815,97 | | 30593,12 | |  |
|  | LOS | 1474 | | 23,09 | | 21,28 | | 10,00 | | 17,00 | | 29,00 | | 47,00 | |  |
|  |  |  | |  | |  | |  | |  | |  | |  | |  |
| 01M30T | Total cost | 1385 | | 1450,90 | | 1180,38 | | 809,39 | | 1037,16 | | 1528,35 | | 2865,76 | |  |
|  | LOS | 1385 | | 0,50 | | 0,50 | | 0,00 | | 0,00 | | 1,00 | | 1,00 | |  |
|  |  |  | |  | |  | |  | |  | |  | |  | |  |
| 01M311 | Total cost | 263 | | 3623,44 | | 3028,49 | | 1550,92 | | 2523,99 | | 4542,65 | | 7781,53 | |  |
|  | LOS | 263 | | 4,40 | | 3,72 | | 2,00 | | 3,00 | | 6,00 | | 9,00 | |  |
|  |  |  | |  | |  | |  | |  | |  | |  | |  |
| 01M312 | Total cost | 273 | | 6544,21 | | 15575,11 | | 2868,15 | | 4458,46 | | 7017,82 | | 10495,03 | |  |
|  | LOS | 273 | | 8,59 | | 5,91 | | 4,00 | | 7,00 | | 11,00 | | 15,00 | |  |
|  |  |  | |  | |  | |  | |  | |  | |  | |  |
| 01M313 | Total cost | 237 | | 8304,93 | | 8196,07 | | 4031,26 | | 6578,69 | | 9482,74 | | 15116,37 | |  |
|  | LOS | 237 | | 14,53 | | 19,46 | | 7,00 | | 11,00 | | 17,00 | | 26,00 | |  |
|  |  |  | |  | |  | |  | |  | |  | |  | |  |
| 01M314 | Total cost | 98 | | 12288,67 | | 9543,02 | | 5857,45 | | 9989,01 | | 14334,08 | | 25728,27 | |  |
|  | LOS | 98 | | 17,13 | | 12,87 | | 8,00 | | 13,50 | | 22,00 | | 34,00 | |  |
|  |  |  | |  | |  | |  | |  | |  | |  | |  |
| 01M31T | Total cost | 465 | | 1275,78 | | 1127,59 | | 667,94 | | 885,03 | | 1308,04 | | 2687,77 | |  |
|  | LOS | 465 | | 0,46 | | 0,50 | | 0,00 | | 0,00 | | 1,00 | | 1,00 | |  |

Tableau A8 _ Stroke, Year 2019, **Private Sector**: Detailed information on Cost and LOS per French-DRG

| DRG-Root | Variable | | N | | Mean | | Std. Dev. | | Q25 | | Q50 | | Q75 | | D90 | |
| --- | --- | --- | --- | --- | --- | --- | --- | --- | --- | --- | --- | --- | --- | --- | --- | --- |
|  |  |  | |  | |  | |  | |  | |  | |  | |  |
| 01M30 | Total cost | 274 | | 4983,64 | | 6234,42 | | 1665,77 | | 3190,96 | | 5346,25 | | 11160,37 | |  |
|  | LOS | 274 | | 11,21 | | 16,41 | | 3,00 | | 7,00 | | 13,00 | | 25,00 | |  |
|  |  |  | |  | |  | |  | |  | |  | |  | |  |
| 01M31 | Total cost | 61 | | 4759,11 | | 13467,72 | | 671,54 | | 1732,65 | | 4338,67 | | 6906,84 | |  |
|  | LOS | 61 | | 11,25 | | 41,57 | | 0,00 | | 3,00 | | 9,00 | | 13,00 | |  |
|  |  |  | |  | |  | |  | |  | |  | |  | |  |
| DRG-Root | Variable | | N | | Mean | | Std. Dev. | | Q25 | | Q50 | | Q75 | | D90 | |
|  |  |  | |  | |  | |  | |  | |  | |  | |  |
| 01M301 | Total cost | 79 | | 2587,18 | | 1297,71 | | 1537,93 | | 2228,59 | | 3459,93 | | 4701,10 | |  |
|  | LOS | 79 | | 5,65 | | 3,56 | | 3,00 | | 5,00 | | 8,00 | | 12,00 | |  |
|  |  |  | |  | |  | |  | |  | |  | |  | |  |
| 01M302 | Total cost | 60 | | 4024,37 | | 2098,80 | | 2583,50 | | 3655,92 | | 4927,52 | | 6673,37 | |  |
|  | LOS | 60 | | 8,67 | | 5,11 | | 5,00 | | 8,00 | | 10,00 | | 15,50 | |  |
|  |  |  | |  | |  | |  | |  | |  | |  | |  |
| 01M303 | Total cost | 69 | | 8453,03 | | 8727,29 | | 3446,79 | | 5609,23 | | 10854,60 | | 17749,19 | |  |
|  | LOS | 69 | | 20,72 | | 25,08 | | 7,00 | | 13,00 | | 24,00 | | 40,00 | |  |
|  |  |  | |  | |  | |  | |  | |  | |  | |  |
| 01M304 | Total cost | 25 | | 12221,36 | | 8301,07 | | 5167,48 | | 9690,45 | | 21304,38 | | 24294,58 | |  |
|  | LOS | 25 | | 26,20 | | 19,14 | | 14,00 | | 18,00 | | 33,00 | | 65,00 | |  |
|  |  |  | |  | |  | |  | |  | |  | |  | |  |
| 01M30T | Total cost | 41 | | 753,05 | | 308,17 | | 614,17 | | 688,12 | | 793,04 | | 1144,92 | |  |
|  | LOS | 41 | | 0,51 | | 0,51 | | 0,00 | | 1,00 | | 1,00 | | 1,00 | |  |
|  |  |  | |  | |  | |  | |  | |  | |  | |  |
| 01M311 | Total cost | 8 | | 1596,56 | | 604,62 | | 1126,85 | | 1430,50 | | 2088,75 | | 2516,99 | |  |
|  | LOS | 8 | | 3,00 | | 1,41 | | 2,00 | | 2,50 | | 3,50 | | 6,00 | |  |
|  |  |  | |  | |  | |  | |  | |  | |  | |  |
| 01M312 | Total cost | 11 | | 4143,60 | | 1657,06 | | 3527,27 | | 4512,48 | | 4844,04 | | 5779,74 | |  |
|  | LOS | 11 | | 10,18 | | 4,85 | | 5,00 | | 11,00 | | 13,00 | | 13,00 | |  |
|  |  |  | |  | |  | |  | |  | |  | |  | |  |
| 01M313 | Total cost | 13 | | 14770,63 | | 27364,04 | | 3384,61 | | 5696,03 | | 8278,73 | | 25028,25 | |  |
|  | LOS | 13 | | 38,92 | | 86,43 | | 7,00 | | 9,00 | | 20,00 | | 74,00 | |  |
|  |  |  | |  | |  | |  | |  | |  | |  | |  |
| 01M314 | Total cost | 3 | | 6394,64 | | 5244,01 | | 2490,14 | | 4338,67 | | 12355,10 | | 12355,10 | |  |
|  | LOS | 3 | | 12,67 | | 8,14 | | 7,00 | | 9,00 | | 22,00 | | 22,00 | |  |
|  |  |  | |  | |  | |  | |  | |  | |  | |  |
| 01M31T | Total cost | 26 | | 798,14 | | 604,54 | | 440,83 | | 625,89 | | 860,62 | | 1891,51 | |  |
|  | LOS | 26 | | 0,23 | | 0,43 | | 0,00 | | 0,00 | | 0,00 | | 1,00 | |  |

# APPENDIX B

Figure B1 : Number of French-DRGs

| \|  \| \| --- \|   *Source:* Book. “Economie de la santé et des systèmes de santé”, 2024. ed. Ellipses. |
| --- | --- |

Figure B2: Length of Stay (LOS) variation by disorder and DRG

|  |  |
| --- | --- |
|  |  |

Note : Each code represents a DRG, which stands for the root associated with a specific severity level. The mean is represented by a dot, and the standard deviation is shown by a vertical segment that defines the interval.

Figure B3: box-and-whisker plot for THR, DRG-root : 08C47

Figure B4: box-and-whisker plot for THR, DRG-root : 08C48

Figure B5: box-and-whisker plot for Stroke, DRG-root : 01M30

Figure B6: box-and-whisker plot for Stroke, DRG-root : 01M31

Figure B7: box-and-whisker plot for Cataract, DRG-root : 02C05

Figure B8: box-and-whisker plot for Cataract, DRG-root : 02C12

Figure B9: box-and-whisker plot for AMI, DRG-root : 05M04

Figure B10: box-and-whisker plot for AMI, DRG-root : 05M16

Figure B11: box-and-whisker plot for AMI, DRG-root : 05K05

Figure B12: box-and-whisker plot for AMI, DRG-root : 05K06

Figure B13: box-and-whisker plot for AMI, DRG-root : 05K21
